# Mixed Amphiphilic Polymeric Nanoparticles of Chitosan, Poly(vinyl alcohol) and Poly(methyl methacrylate) for Intranasal Drug Delivery: A Preliminary In Vivo Study

**DOI:** 10.3390/molecules25194496

**Published:** 2020-09-30

**Authors:** Inbar Schlachet, Hen Moshe Halamish, Alejandro Sosnik

**Affiliations:** Laboratory of Pharmaceutical Nanomaterials Science, Department of Materials Science and Engineering, Technion-Israel Institute of Technology, Technion City, Haifa 3200003, Israel; inbarschlachet@gmail.com (I.S.); chen.moshe.num1@gmail.com (H.M.H.)

**Keywords:** central nervous system (CNS), blood–brain barrier (BBB), self-assembled polymeric nanoparticles, intranasal delivery, biodistribution

## Abstract

Intranasal (i.n.) administration became an alternative strategy to bypass the blood–brain barrier and improve drug bioavailability in the brain. The main goal of this work was to preliminarily study the biodistribution of mixed amphiphilic mucoadhesive nanoparticles made of chitosan-*g*-poly(methyl methacrylate) and poly(vinyl alcohol)-*g*-poly(methyl methacrylate) and ionotropically crosslinked with sodium tripolyphosphate in the brain after intravenous (i.v.) and i.n. administration to Hsd:ICR mice. After i.v. administration, the highest nanoparticle accumulation was detected in the liver, among other peripheral organs. After i.n. administration of a 10-times smaller nanoparticle dose, the accumulation of the nanoparticles in off-target organs was much lower than after i.v. injection. In particular, the accumulation of the nanoparticles in the liver was 20 times lower than by i.v. When brains were analyzed separately, intravenously administered nanoparticles accumulated mainly in the “top” brain, reaching a maximum after 1 h. Conversely, in i.n. administration, nanoparticles were detected in the “bottom” brain and the head (maximum reached after 2 h) owing to their retention in the nasal mucosa and could serve as a reservoir from which the drug is released and transported to the brain over time. Overall, results indicate that i.n. nanoparticles reach similar brain bioavailability, though with a 10-fold smaller dose, and accumulate in off-target organs to a more limited extent and only after redistribution through the systemic circulation. At the same time, both administration routes seem to lead to differential accumulation in brain regions, and thus, they could be beneficial in the treatment of different medical conditions.

## 1. Introduction

The treatment of diseases of the central nervous system (CNS) by systemic drug administration is challenging, owing to the presence of the blood–brain barrier (BBB) and the blood–cerebrospinal fluid barrier [1]. The BBB excludes more than 95% of the small-molecule and biological drugs from crossing into the brain [2,3]. In addition, the BBB displays different efflux transporters that transport substrate molecules (e.g., drugs) out of the brain endothelium, against a concentration gradient [4,5]. Drugs that do not comply with fundamental physicochemical characteristics such as high lipid solubility, low molecular weight and less than 8–10 H bonds with water cannot cross the BBB and their bioavailability and pharmacological efficacy diminished [2].

New delivery approaches that increase drug delivery to the CNS are under intense investigation [6]. The transient disruption of the BBB by osmotic shrinkage of the endothelial cells together with the opening of BBB tight junctions by intracarotid arterial infusion of non-diffusive solutes such as mannitol is one of them [7]. A main drawback is that increased permeability might also enable the passage of plasma proteins and result in abnormal neuronal function [8]. Another strategy is the use of carrier-mediated transport systems that transport nutrients such as glucose and amino acids into the CNS [8]. Drugs with the proper molecular design that do not always comply with the structure–activity relationship can be recognized by these influx transporters and show high permeability across the BBB [2,8,9]. Over recent decades, a plethora of nanotechnology strategies have been investigated to overcome the limited ability to deliver active molecules from the systemic circulation into the CNS [10,11,12,13,14,15]. For this, drug-loaded nanoparticles of a different nature (e.g., lipid, polymeric) and size are surface-decorated with ligands that bind receptors overexpressed in the BBB and cross the BBB by transcytosis [16,17].

The existence of a nose-to-brain pathway that bypasses the BBB has been evidenced by the accumulation and harm caused by environmental nanoparticulate matter in the CNS [18,19,20,21]. With the emergence of nanomedicine, different types of pure drug nanocrystals and nanoparticles were designed and their CNS bioavailability following intranasal (i.n.) administration assessed [22,23,24,25,26]. Recent studies confirmed the advantage of i.n. administration of nanocarriers over the intravenous (i.v.) route to increase drug bioavailability in the olfactory bulb. Among the different nanotechnology delivery platforms, polymeric micelles are among the most promising [22]. For example, in an early study, we demonstrated that the i.n. administration of the antiretroviral efavirenz nanoencapsulated within core-corona polymeric micelles made of poly(ethylene oxide)-*b*-poly(propylene oxide) (PEO-PPO) block copolymers significantly increases its bioavailability in the brain of rats with respect to the i.v. counterpart [27]. In this context, fundamental nanoparticle features (e.g., size, shape, surface chemistry) that govern the nose-to-brain transport remain to be elucidated [28]. For instance, the size, the shape and/or the surface chemistry and charge could change the transport pathway.

PEO-PPO polymeric micelles show limited encapsulation capacity for many hydrophobic cargos and low physical stability upon dilution over time. In this context, we developed mucoadhesive amphiphilic polymeric nanoparticles produced by the aggregation of chitosan (CS) and poly(vinyl alcohol) (PVA) graft copolymers hydrophobized in the side-chain with different hydrophobic blocks such as poly(methyl methacrylate) (PMMA) and their physical stabilization by non-covalent crosslinking of CS and PVA domains with sodium tripolyphosphate (TPP) and boric acid, respectively [29,30,31,32,33]. These nanoparticles display a multimicellar nanostructure [32,33] and very high physical stability under extreme dilution [29,30,31,32,33]. Aiming to understand the cellular pathways involved in this transport, we recently investigated the interaction of these polymeric nanoparticles with primary olfactory sensory neurons, cortical neurons and microglia isolated from olfactory bulb, olfactory epithelium and cortex of newborn rats [34]. Our results strongly suggested the involvement of microglia (and not cortical or olfactory neurons) in the nose-to-brain transport of nanoparticulate matter.

CS has been extensively investigated as a mucoadhesive drug nanocarrier, and its cytotoxicity is a matter of debate [35,36], including for i.n. drug delivery [37,38,39,40]. Most works reported on the good cell compatibility of this polysaccharide that is classified as “generally recognized as safe” (GRAS) by the US Food and Drug Administration (FDA) [41]. However, CS nanoparticles have been also associated with cell toxicity because of the electrostatic interaction of the positively-charged surface with the negatively-charged cell membrane, and the toxicity level depends on the cell type [42].

Aiming to capitalize on the potential of our versatile amphiphilic nanocarriers in mucosal drug delivery in general and in nose-to-brain administration in particular, we preliminary investigated the cell compatibility of CS-*g*-PMMA nanoparticles in human primary nasal epithelial cells and showed their high toxicity [42]. In this context, we produced mixed CS-*g*-PMMA:PVA-*g*-PMMA (1:1 weight ratio) nanoparticles that display better human nasal cell compatibility than the CS-based counterparts owing to a decrease in the surface charge density, as expressed by a less positive zeta-potential (Z-potential). In addition, we demonstrated that they cross a model of nasal epithelium in vitro [43]. These nanoparticles encapsulated two experimental anticancer drugs [43]. In this work, we preliminarily investigated the biodistribution of these mixed amphiphilic nanoparticles after i.n. administration to Hsd:ICR mice and compared it to the i.v. route for the first time.

## 2. Results and Discussion

Mixed nanoparticles were produced by the solvent casting method that comprised co-dissolution of identical amounts of CS-PMMA30 (a CS-*g*-PMMA copolymer containing 30% *w*/*w* of PMMA) and PVA-PMMA16 (a PVA-*g*-PMMA copolymer containing 16% *w*/*w* of PMMA) in dimethyl sulfoxide (DMSO), drying under vacuum and redispersion in water [43,44]. Self-assembly takes place once the critical aggregation concentration (CAC) is surpassed. The CAC of CS-PMMA30 and PVA-PMMA16 is in the 0.04–0.05% *w*/*v* range [30,31,32,33]. Since the self-assembly process is random, by utilizing this method, we anticipated the formation of mixed nanoparticles with very similar qualitative and quantitative composition. To physically stabilize the nanoparticle, CS domains were crosslinked by the formation of a polyelectrolyte complex with TPP. The size, size distribution and Z-potential of 0.1% *w*/*v* non-crosslinked and TPP-crosslinked mixed CS-PMMA30:PVA-PMMA16 nanoparticles before the in vivo studies were analyzed by dynamic light scattering (DLS), at 25 °C [42]. Non-crosslinked and crosslinked nanoparticles showed monomodal size distribution (one size population), while the polydispersity index (PDI), which is a measure of the size distribution, slightly changed after the ionotropic crosslinking; e.g., non-crosslinked mixed CS-PMMA30:PVA-PMMA16 nanoparticles showed a hydrodynamic diameter (D_h_) of 193 ± 62 nm and a PDI of 0.23 (Table S1) [42]. This size is similar to that shown by pure CS-PMM30 (D_h_ of 184 ± 4 nm; PDI of 0.20) nanoparticles and larger than that of pure PVA-PMMA16 counterparts of the same concentration (D_h_ of 92 ± 4 nm and PDI of 0.14) [33]. Crosslinking of a 0.1% *w*/*v* nanoparticle suspension with TPP solution in water (1% *w*/*v*; 2.5 µL per mL of nanoparticles) resulted in an increase in the size to 249 ± 26 nm and in the PDI to 0.26, and their full physical stabilization [42]. Pure crosslinked CS-PMM30 nanoparticles are larger—332 ± 54 nm (PDI of 0.33)—owing to nanoparticle bridging [32], a phenomenon that is less likely in mixed particles that contain 50% *w*/*w* of non-ionic PVA-PPMA16, a copolymer that does not interact with TPP.

The surface charge of nanoparticulate matter affects their cell compatibility, and usually, positively-charged particles are more cytotoxic than neutral and negatively-charged ones [45]. CS has been extensively reported as a biocompatible polysaccharide, and it is approved in the food industry [41]. However, it may elicit cell toxicity in vitro due to a highly positively-charged surface [36,37,46]. Crosslinking of self-assembled CS-based nanoparticles was implemented to physically stabilize them and to partly neutralize the net positive surface charge and increase their cell compatibility [30]. This modification was not enough to ensure their good compatibility with human primary nasal epithelium cells [42]. Thus, we produced mixed nanoparticles that reduce the effective CS concentration on the surface and thus, its charge density, while preserving the nanoencapsulation capacity of the nanoparticles and its mucoadhesiveness [42]. Further crosslinking reduced the Z-potential and improved the compatibility of the nanoparticles in primary nasal epithelial cells [42,43]. 

**Figure 1:**
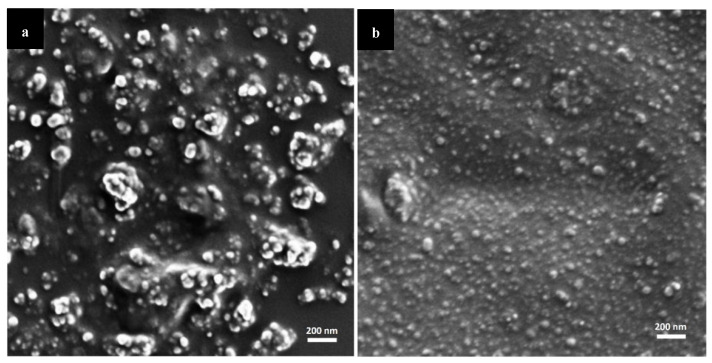
HR-SEM micrographs of mixed CS-*g*-PMMA:PVA-*g*-PMMA nanoparticles. (**a**) non-crosslinked and (**b**) TPP-crosslinked nanoparticles.

We visualized the morphology of non-crosslinked and crosslinked nanoparticles by high resolution-scanning electron microscopy (HR-SEM). The size was in line with DLS analysis, considering that in HR-SEM, the nanoparticles underwent drying as opposed to DLS where the D_h_ is measured (Figure 1). Some aggregation during sample preparation could not be prevented, though these aggregates are not present in the nanoparticle suspension.

**Figure 2:**
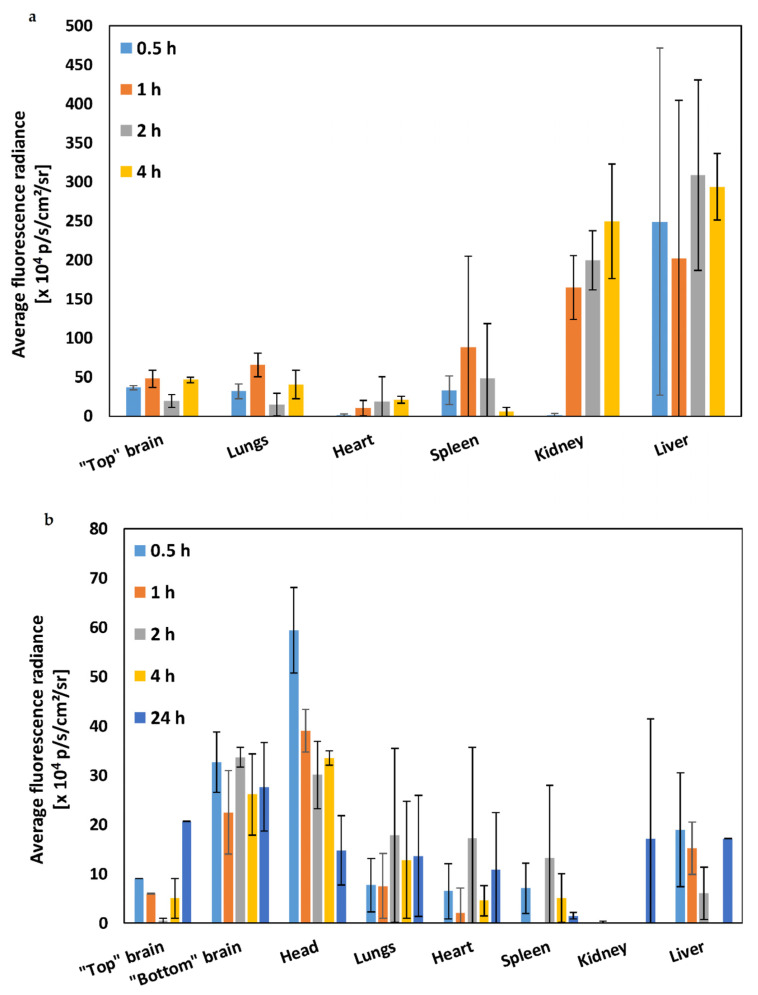
Biodistribution of NIR-797-labeled crosslinked 0.1% w/v mixed CS-PMMA30:PVA-PMMA16 nanoparticles after (**a**) i.v. administration and (**b**) i.n. administration to Hsd:ICR mice (n = 3). The measurement was performed after organ dissection at each time point. Average fluorescence radiance was measured using Living Imaging analysis software. Bars represent the average of mice at each time point. The error bars are S.D. from the mean. Statistical comparisons are summarized in Table S2.

Since in a previous work, we showed that these nanoparticles cross a model of the human nasal epithelium in vitro [42], we hypothesized that they could effectively reach the CNS upon i.n. administration. In this framework, the main goal of this work was to investigate for the first time the biodistribution and accumulation in the brain and other organs of mixed CS-PMMA30:PVA-PMMA16 nanoparticles after i.n. administration to Hsd:ICR mice and compare it to the i.v. route. Since crosslinked nanoparticles are physically stable, as opposed to the non-crosslinked counterparts, for this preliminary study, 0.1% *w*/*v* crosslinked mixed CS-PMMA30:PVA-PMMA16 nanoparticles were labeled with the near infrared (NIR) dye NIR-797 and 200 µL of the nanoparticle suspension was injected i.v. through the tail vein (total nanoparticle dose of 8 mg/kg), or 20 µL of the same formulation was administered i.n. (total nanoparticle dose of 0.8 mg/kg). It is important to highlight that in this preliminary study, the nanoparticle dose administered i.n. was 10-fold smaller than i.v. At predetermined time points, live animal screening was performed using IVIS Spectrum In Vivo Imaging System. After i.v. administration, nanoparticles reach the systemic circulation and interact with the reticuloendothelial system, a system of macrophages mostly in the liver that could sequester the nanoparticles due the recognition of opsonins (serum proteins), while nanoparticles with size of up to 5–10 nm could undergo renal filtration [47]. At different time points post-administration (0–24 h), mice were sacrificed, the different organs carefully dissected to prevent cross contamination and the average fluorescence radiance (AFR) of each organ was quantified by subtracting the basal signal of each organ in control (untreated) animals (Figure 2). After i.v. administration, the highest accumulation at the different time points was observed in the liver (Figure 2a), as described for other nanoparticles of similar size and composition upon i.v. administration [48,49,50]. Other organs showed lower AFR associated with a more limited nanoparticle off-target accumulation. According to the size (several hundreds of nanometers), these nanoparticles do not undergo renal filtration. Thus, their detection in the kidneys is most probably related to their accumulation in the renal tissue (e.g., proximal tube epithelium) [50].

Intranasal is a local administration route that capitalizes on the nose-to-brain transport to surpass the BBB and target different parts of the brain. Thus, accumulation in peripheral organs such as the liver was expected to take place to a very limited extent [51]. After i.n. administration, the accumulation of the nanoparticles in off-target organs was much lower than after i.v. injection (Figure 2b). Moreover, a comparison of the AFR values in the different organs at different time points (0–4 h) after i.n. and i.v. administration revealed that some of the differences between both administration routes were statistically significant (Table S2). In particular, the accumulation of the nanoparticles in the liver, which is the main clearance organ for nanoparticulate matter in this size range, was up to 20 times lower after i.n. administration than by i.v. even though the dose was 10 times smaller; intranasally administered nanoparticles could reach peripheral organs after redistribution from the CNS to the systemic circulation [52,53]. These results highlight the benefit of i.n. administration to reduce off-target delivery and toxicity.

**Figure 3:**
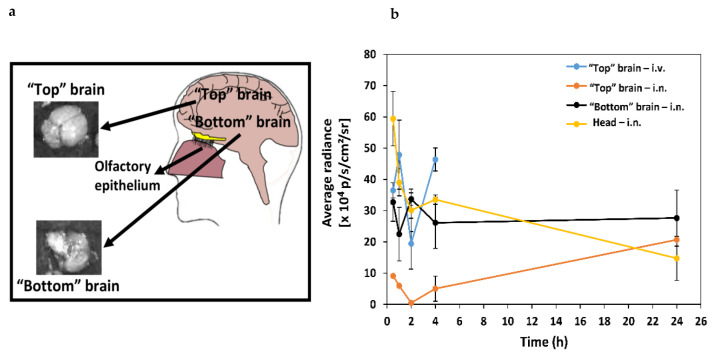
Ex vivo analysis of the distribution of NIR-797-labeled crosslinked 0.1% *w*/*v* mixed CS-PMMA30: PVA-PMMA16 nanoparticles in the brain following i.v. and i.n. administration to Hsd:ICR mice (n = 3). (**a**) Scheme of the top and bottom brain and (**b**) average radiance over time obtained after the subtraction of the control (untreated mice brain) radiance (n = 3).

The imaging system used in this study normalizes the AFR to the organ that displays the maximum intensity, in this case the liver. Thus, we imaged the brains separately from the other organs (in triplicates) at different time points (0–24 h, depending on the administration route) and estimated the nanoparticle accumulation in the “top” brain (i.v. and i.n. administration), and “bottom” brain and head (i.n. administration) (Figure 3a). Upon i.v. administration (0–4 h), our nanoparticles accumulated mainly in the “top” brain. Later time points were not investigated in this preliminary study because we previously showed the relatively limited bioavailability of this type of nanoparticle in the CNS of mice after i.v. administration and the need for the surface modification with ligands that bind receptors expressed on the apical side of the BBB endothelium [49]. We were more interested in exploring the behavior of intranasally administered nanoparticles, and thus, we tracked them for 24 h. Different i.n. administration methods and formulations could affect the biodistribution of the drug-loaded nanoparticles in the CNS and the pharmacological outcome. For example, Martins et al. showed that the i.n. administration of oxytocin with a nebulizer leads to a different pharmacological outcome compared to a standard nasal spray [54]. These results highlight the complexity of this transport pathway and the difficulty of comparing among works that used different formulations, doses and administration regimens. After i.n. administration, particles are expected to enter the CNS through the olfactory region and accumulate in the nose, the nose-to-brain tract (e.g., olfactory bulb) and the “bottom” brain (Figure 3a), before they disseminate to all the brain [21,55].

The highest AFR value was measured in the “top” brain 1 h after i.v. injection (Figure 3b). At this point, we analyzed the bottom side of the brain after i.n. administration because after penetrating through the olfactory epithelium, the nanoparticles could be accumulated in this area of the CNS close to the pons and serve as a reservoir from which an encapsulated drug could be released. Two hours after i.n. administration, the accumulation in the “bottom” brain was significantly higher than upon i.v. injection. We further calculated the AFR in the brain (with subtraction of the control signal) at each time point and compared values of area under the curve (AUC) between 0 and 4 h (AUC_0–4 h_). Nanoparticle accumulation in the “bottom” brain after i.n. administration (AUC_0–4 h_ = 110 ± 10 × 10^4^ p/s/cm^2^/sr) was similar and not significantly different from that of intravenously administered nanoparticles in the “top” brain (AUC_0–4 h_ = 130 ± 20 × 10^4^ p/s/cm^2^/sr) (Figure 3b, Table 1). These results indicate that a similar brain bioavailability could be reached with a 10 times smaller dose. At the same time, it is important to point out that in the case of i.n. administration, these mucoadhesive nanoparticles could be initially retained in the nasal mucosa and accumulate in the nose-to-brain tract and, at a later stage, be released and diffuse across the brain tissue to reach more distant areas.

In general, two beneficial phenomena were observed after i.n. administration when compared to i.v.: (i) there was higher accumulation in the brain [51] and (ii) the accumulation was less spread, enabling targeting of the nanoparticles to more specific CNS regions, which is associated with the nanoparticle properties [56] and probably with the type of nasal nanoformulation [54]. After i.n. administration, our mucoadhesive nanoparticles reached the brain quickly (less than 1 h) and could be detected mainly in the head due to their retention in the nose and accumulated mainly in the “bottom” brain, while the AFR in the “top” brain was relatively low. The fast delivery of nanoparticles to the brain upon i.n. administration has been reported in the literature [57,58]. It is also important to stress that nose tissues were not isolated, and thus, the whole head without the brain was imaged to estimate the retention of the nanoparticles in the nasal mucosa. The AUC_0–4 h_ in the “bottom” brain was significantly higher than that in the “top” brain, indicating that the nanoparticles initially accumulate in the nose-brain tract (Figure 3b, Table 1). At 24 h, the AFR in the “bottom” brain remained almost constant (AFR ratio between 24 and 4 h was 1.06) and it increased by 4-fold in the “top” brain with respect to 4 h, at the expense of the AFR detected in the head/nose. A similar trend was followed by the AUC_0–24 h_ that showed a moderate increase in the “bottom” brain from 110 ± 18 × 10^4^ to 164 ± 7 × 10^4^ p/s/cm^2^/sr and a more pronounced one in the “top” brain from 15 ± 4 × 10^4^ to 41 ± 3 × 10^4^ p/s/cm^2^/sr (Table 1). These findings suggest that the nanoparticles are transported from the nasal mucosa and the “bottom” brain to other brain areas at a slower rate, leading to these changes in the AUC_0–24 h_ values. Most previous research utilizing i.n. delivery of nanomedicines disregarded the possible differential biodistribution and assumed that all the brain areas are exposed to similar nanoparticle concentrations. Our results with mixed CS-PMMA30:PVA-PMMA16 nanoparticles strongly suggest that they are not homogeneously distributed in the brain soon after administration. In addition, they strongly suggest that with the proper nanoparticle design, specific structures of the CNS could be targeted to treat different medical conditions affecting them.

Having said this, more studies need to be conducted to realize this potential. Future studies will investigate the pharmacokinetics of encapsulated drugs in the CNS upon i.v. and i.n. administration and will also include later time points.

## 3. Methods

### 3.1. Synthesis of the Chitosan-g-Poly(methyl methacrylate) and Poly(vinyl alcohol)-g-Poly(methyl methacrylate) Copolymers

A CS-*g*-PMMA copolymer containing 30% *w*/*w* of PMMA (CS-PMMA30, as determined by proton-nuclear magnetic resonance [31,32]) was synthesized by the graft free radical polymerization of MMA (99% purity, Alfa Aesar, Heysham, UK) onto the CS backbone in water. For this, low molecular weight CS (0.4 g, degree of deacetylation of 94%; viscosity ≤100 mPa.s, Glentham Life Sciences, Corsham, UK) was dissolved in nitric acid 70% (0.05 M in water, 100 mL) that was degassed by sonication (30 min, Elmasonic S 30, Elma Schmidbauer GmbH, Singen, Germany). Then, a tetramethylethylenediamine (TEMED, Alfa Aesar) solution (0.18 mL in 50 mL degassed water) was poured into the CS solution and purged with nitrogen for 30 min at room temperature (RT). The purged CS solution was magnetically stirred and heated to 35 °C, and 142 µL MMA (distilled under vacuum to remove inhibitors before use) was added to the degassed water (48 mL) and then mixed with the CS solution. Finally, a cerium (IV) ammonium nitrate (CAN, Strem Chemicals, Inc., Newburyport, MA, USA) solution (0.66 g in 2 mL degassed water) was added to the polymerization reaction that was allowed to proceed for 3 h at 35 °C and under continuous nitrogen flow. After 3 h, the polymerization was quenched by adding 0.13 g of hydroquinone (Merck, Hohenbrunn, Germany). The product was purified by dialysis against distilled water using a regenerated cellulose dialysis membrane with a molecular weight cut-off (MWCO) of 12–14 kDa (Spectra/Por^®^ 4 nominal flat width of 75 mm, diameter of 48 mm and volume/length ratio of 18 mL/cm; Spectrum Laboratories, Inc., Rancho Dominguez, CA, USA) for 48–72 h and freeze-dried. The same chemical pathway with small modifications was used to synthesize a PVA-*g*-PMMA copolymer containing 16% *w*/*w* of PMMA (PVA-PMMA16) [33]. First, PVA (0.4 g) was dissolved in distilled water (100 mL) at RT, and TEMED (0.18 mL in 50 mL degassed water) was dissolved in 70% nitric acid (0.45 mL). Then, TEMED and PVA solutions were degassed by sonication for 30 min, mixed and purged with nitrogen for 30 min at RT. The solution was heated to 35 °C and 142 µL MMA dispersed in degassed water (48 mL) and added to the reaction mixture. Finally, a CAN solution (0.66 g in 2 mL degassed water) was added and the reaction allowed to proceed for 2 h at 35 °C. The reaction product was purified by dialysis and freeze-dried. Products were stored at 4 °C until use.

For biodistribution studies (see below), CS-PMMA30 and PVA-PMMA16 copolymers were fluorescently-labeled with the near infrared tracer NIR-797 isothiocyanate (Sigma-Aldrich, St. Louis, MO, USA). For this, CS-PMMA30 (80 mg) was dissolved in 8 mL water supplemented with acetic acid (pH = 5.5), prepared by diluting 70 µL glacial acetic acid (Bio-Lab Ltd., Jerusalem, Israel) in 1 L water under magnetic stirring. Then, NIR-797 (0.4 mg) was dissolved in *N*,*N*-dimethylformamide (0.2 mL, DMF, Bio-Lab Ltd.), added to the copolymer solution and the mixture stirred for 16 h protected from light, at RT. Finally, the product was dialyzed (48 h, regenerated cellulose dialysis membrane, MWCO of 3500 Da, Membrane Filtration Products, Inc., Seguin, TX, USA), freeze-dried (72–96 h) and stored protected from light at 4 °C until use. In the case of PVA-PMMA16, the copolymer (100 mg) was dissolved in 3 mL DMF under magnetic stirring. Then, NIR-797 (0.8 mg) was dissolved in DMF (0.1 mL), added to the copolymer solution and the mixture stirred for 16 h protected from light, at RT. The reaction mixture was diluted with deionized water (1:2 *v*/*v*), dialyzed (48 h, regenerated cellulose dialysis membrane, MWCO of 3500 Da) to remove unreacted NIR-797, freeze-dried (72–96 h) and stored protected from light at 4 °C until use. The theoretical NIR-797 content was between 0.5 and 0.8% *w*/*w*.

### 3.2. Preparation and Characterization of Mixed Chitosan-g-Poly(methyl methacrylate):Poly(vinyl alcohol)-g-Poly(methyl methacrylate) Nanoparticles

Identical amounts of CS-PMMA30 and PVA-PMMA16 were dissolved in DMSO to reach a total copolymer concentration of 0.5% *w*/*v* under continuous stirring (24 h), at 37 °C. Subsequently, the solution was dried under vacuum utilizing a freeze-dryer, the copolymer mixture re-dispersed in water supplemented with acetic acid (pH = 5.5) to reach a final total copolymer concentration of 0.1% *w*/*v* and filtered (1.2 µm cellulose acetate syringe filter, Sartorius Stedim Biotech GmbH, Göttingen, Germany). For physical stabilization, 0.1% *w*/*v* nanoparticles were crosslinked by the addition of 1% *w*/*v* TPP (Sigma-Aldrich) aqueous solution (2.5 µL of crosslinking solution per mL of 0.1% *w*/*v* nanoparticle dispersion).

The size (expressed as hydrodynamic diameter, D_h_), size distribution (estimated by the PDI) and the Z-potential (an estimation of the surface charge density) of 0.1% *w*/*v* systems were measured using the Zetasizer Nano-ZS in the same media detailed above for the different samples. Z-potential measurements of the same samples required the use of laser Doppler microelectrophoresis in the Zetasizer Nano-ZS. Each value obtained is expressed as the mean ± standard deviation (S.D.) of at least three independent samples, while each DLS or Z-potential measurement is an average of at least seven runs.

The morphology of mixed nanoparticles before and after crosslinking was visualized by HR-SEM (carbon coating, acceleration voltage of 2–4 kV, Ultraplus, Zeiss, Oberkochen, Germany). For this, mixed nanoparticle suspensions (0.5% *w*/*v* total copolymer concentration) were drop-casted on silicon wafer, dried at 37 °C in the oven and carbon-coated. Images were obtained using an in-lens detector at 3–4 mm working distance. The nanosuspensions were sprayed on top of a silicon wafer (cz polished silicon wafers <100> oriented, highly doped N/Arsenic, SHE Europe Ltd., Livingston, UK) by introducing high pressure nitrogen which allowed an even spread of the nanoparticles on the wafer. Next, the wafer was attached to the grid using carbon-tape, and additional tape was placed on its frame. At the corners of the frame, silver paint (SPI# 05002-AB—Silver, SPI supplies, West Chester, PA, USA) was applied and the samples were carbon coated.

### 3.3. Biodistribution of Mixed Chitosan-g-Poly(methyl methacrylate):poly(vinyl alcohol)-g-Poly(methyl methacrylate) Nanoparticles

For biodistribution studies, CS-PMMA30 and PVA-PMMA16 copolymers were fluorescently labeled with NIR-797, as described above.

Hsd:ICR mice (Envigo, Jerusalem, Israel) were maintained at the Gutwirth animal facility of the Technion-Israel Institute of Technology. All animal experiments were approved and performed according to the guidelines of the Institutional Animal Research Ethical Committee at the Technion (ethics approval number IL-052-05-18). Animal welfare was monitored daily by the staff veterinarians. Mice fasted for 12 h prior to experiments. Crosslinked mixed CS-PMMA30:PVA-PMMA16 nanoparticles (200 µL, 0.1% *w*/*v*) were injected i.v. into the tail vein. For i.n. administration, mice were lightly anesthetized with 2.5% isoflurane (USP Terrel™ Piramal Critical Care, Bethlehem, PA, USA), and fixed in a supine position for the administration of 10 µL of the nanoparticles in each nostril using a pipette (total volume of 20 µL, 0.1% *w*/*v*). After 0.5, 1, 2, 4 and 24 h post-i.v. injection or i.n. administration, animals were sacrificed by dislocation and organs (liver, spleen, kidney, lungs, heart, brain, and head/nose) were dissected. Organ screening was performed ex vivo using an Imaging System (IVIS, PerkinElmer, Waltham, MA, USA) with an excitation at 795 nm and an emission at 810 nm. Then, at the same conditions (see above), image analysis was performed using Living Imaging analysis software (PerkinElmer). The auto fluorescence of organs of the control (untreated) mice were subtracted. Mice were used in triplicates for each time point. Then, the average radiance in the brain (with subtraction of the control signal) at each time point was calculated, and the values of AUC_0–4 h_ determined according to Equation (1) [59]

(1) $$\mathrm{AUC}=\underset{i}{\sum }\frac{\left(t_{i+1}-t_{i}\right)}{2\times \left({\mathrm{AR}}_{i}+{\mathrm{AR}}_{i+1}\right)}$$

where t_i_ is the starting time point, t_i+1_ is the finishing time point (0.5, 1, 2 and 4 h), AR_i_ is the starting value of average fluorescence measured and AR_i+1_ is the finishing value for each measurement over time.

### 3.4. Statistical Analysis

Statistical analysis of the different experiments was performed by a *t*-test on raw data (Excel, Microsoft Office 2013, Microsoft Corporation). *P* values smaller than 0.05 were regarded as statistically significant.

## Figures and Tables

**Table 1:** Average fluorescence radiance obtained from different brain regions and the head after the subtraction of the control (untreated mice) radiance at different time points and calculated area under the curve (AUC)_0–4 h_ values.

Brain Region	AUC_0–4 h_ (× 10^4^ p/s/cm^2^/sr) ± S.D.	AUC_0–24 h_ (× 10^4^ p/s/cm^2^/sr) ± S.D.
“Top” brain—i.v.	130 ± 20 *	N.D.
“Top” brain—i.n.	15 ± 4	41 ± 3 *****
“Bottom” brain—i.n.	110 ± 18 **	164 ± 7 **^,^****^,^*****
Head—i.n.	138 ± 17 ***	186 ± 6 *****

N.D.: Not determined. * Statistically significant difference between AUC_0–4 h_ in the “top” brains of mice after i.v. and i.n. administration (*P* < 0.05); ** statistically significant AUC_0–4 h_ and AUC_0–24 h_ difference between the “top” and “bottom” brains of mice after i.n. administration (*P* < 0.05); *** statistically significant AUC_0–4 h_ and AUC_0–24 h_ difference between the “top” brain and the head of mice after i.n. administration (*P* < 0.05); **** statistically significant AUC_0–24 h_ difference between the “bottom” brain and the head of mice after i.n. administration (*P* < 0.05); ***** statistically significant differences between AUC_0–24 h_ and AUC_0–4 h_ after i.n. administration (*P* < 0.05).

## Supplementary Materials

Table S1: hydrodynamic diameter (D_h_), size distribution (PDI) and Z-potential of CS-PMMA30, PVA-PMMA16 and CS-PMMA30:PVA-PMMA16 nanoparticles (total copolymer concentration of 0.1% *w*/*v*), as determined by DLS; Table S2: statistical analysis of AFR data in the different organs and time points after i.v. and i.n. administration of mixed CS-PMMA30:PVA-PMMA16 nanoparticles, as analyzed by the IVIS Spectrum In Vivo Imaging System (*P* < 0.05).
