# Supplementary material for: Mixed Amphiphilic Polymeric Nanoparticles of Chitosan, Poly(vinyl alcohol) and Poly(methyl methacrylate) for Intranasal Drug Delivery: A Preliminary In Vivo Study
Source: Molecules. 2020 Sep 30;25(19):4496. doi: 10.3390/molecules25194496 (PMC7582691; doi:10.3390/molecules25194496)
Supplement: Supplementary file 1 [file molecules-25-04496-s001.pdf]

Supplementary Information

Mixed amphiphilic polymeric nanoparticles of chitosan, poly(vinyl alcohol) and poly(methyl methacrylate) for intranasal drug delivery: A preliminary *in vivo* study

Inbar Schlachet, Hen Moshe Halamish and Alejandro Sosnik[1,*]

[1] Laboratory of Pharmaceutical Nanomaterials Science, Department of Materials Science and Engineering, Technion-Israel Institute of Technology, Haifa, Israel

* Correspondence: Laboratory of Pharmaceutical Nanomaterials Science, De-Jur Bldg., Office 607, Department of Materials Science and Engineering, Technion-Israel Institute of Technology, 3200003 Haifa, Israel; Email: sosnik@technion.ac.il, alesosnik@gmail.com; Tel.: +972-77-887-1971

Supplementary Table 1. Hydrodynamic diameter (D_h), size distribution (expressed as PDI) and Z-potential of CS-PMMA30, PVA-PMMA16 and mixed CS-PMMA30:PVA-PMMA16 nanoparticles (total copolymer concentration of 0.1% w/v), as determined by DLS. Nanoparticle crosslinking was carried out with 1% w/v TPP. Data are reproduced from Refs. 32-34 and 43.

Nanoparticle	TPP volume (μL) [a]	D_h – Intensity (nm) (± S.D.)	PDI (± S.D.)	Z-potential (mV) (± S.D.)
**CS-PMMA30**	-	184 (4)	0.20 (0.01)	+39 (1)
	5	332 (54)	0.33 (0.03)	+28 (2)
**PVA-PMMA16**	-	92 (4)	0.14 (0.01)	-17 (1)
**CS-PMMA30:PVA-PMMA16 (1:1)**	-	193 (62)	0.23 (0.06)	+30 (1)
	2.5	249 (26)	0.26 (0.01)	+10 (1)

[a] Volume of 1% w/v TPP solution per mL of nanoparticle dispersion.

Supplementary Table 2. Statistical analysis of AFR data in the different organs and time points after i.v. and i.n. administration of mixed CS-PMMA30:PVA-PMMA16 nanoparticles, as analyzed by the IVIS Spectrum In Vivo Imaging System (P <0.05).

**Organ**	**Time point**			
	0.5 h	1 h	2 h	4 h
"Top" brain	**Statistically different**	**Statistically different**	**Statistically different**	**Statistically different**
Lungs	**Statistically different**	**Statistically different**	No statistical difference	**Statistically different**
Heart	No statistical difference	No statistical difference	No statistical difference	**Statistically different**
Spleen	No statistical difference	No statistical difference	No statistical difference	No statistical difference
Kidney	No statistical difference	**Statistically different**	**Statistically different**	**Statistically different**
Liver	No statistical difference	No statistical difference	**Statistically different**	**Statistically different**
